# Is antagonistic pleiotropy ubiquitous in aging biology?

**DOI:** 10.1093/emph/eoy033

**Published:** 2018-10-11

**Authors:** Steven N Austad, Jessica M Hoffman

**Affiliations:** Department of Biology, CH464, University of Alabama at Birmingham, Birmingham, AL 35294, USA

**Keywords:** aging, senescence, antagonistic pleiotropy, evolution of aging

## Abstract

Lay Summary: An evolutionary mechanism of aging was hypothesized 60 years ago to be the genetic trade-off between early life fitness and late life mortality. Genetic evidence supporting this hypothesis was unavailable then, but has accumulated recently. These tradeoffs, known as antagonistic pleiotropy, are common, perhaps ubiquitous.

George Williams’ 1957 paper developed the antagonistic pleiotropy hypothesis of aging, which had previously been hinted at by Peter Medawar. Antagonistic pleiotropy, as it applies to aging, hypothesizes that animals possess genes that enhance fitness early in life but diminish it in later life and that such genes can be favored by natural selection because selection is stronger early in life even as they cause the aging phenotype to emerge. No genes of the sort hypothesized by Williams were known 60 years ago, but modern molecular biology has now discovered hundreds of genes that, when their activity is enhanced, suppressed, or turned off, lengthen life and enhance health under laboratory conditions. Does this provide strong support for Williams’ hypothesis? What are the implications of Williams’ hypothesis for the modern goal of medically intervening to enhance and prolong human health? Here we briefly review the current state of knowledge on antagonistic pleiotropy both under wild and laboratory conditions. Overall, whenever antagonistic pleiotropy effects have been seriously investigated, they have been found. However, not all trade-offs are directly between reproduction and longevity as is often assumed. The discovery that antagonistic pleiotropy is common if not ubiquitous implies that a number of molecular mechanisms of aging may be widely shared among organisms and that these mechanisms of aging can be potentially alleviated by targeted interventions.

## INTRODUCTION: THE PUZZLE OF AGING

The logic of evolution by natural selection is straightforward. Within any population, the alleles of individuals that produce the most breeding descendants will increase in frequency in successive generations at the expense of the alleles of individuals less successful at reproduction. To be successful at leaving descendants requires that organisms also be successful at surviving—so that they live long enough to reach reproductive age and afterward continue reproducing. By this logic and process, natural selection ultimately produces individuals superbly designed to survive and reproduce in their environment.

From this perspective, aging presents an evolutionary puzzle. If continued survival and reproduction should always be favored by natural selection, why is aging—which in evolutionary terms can be defined as the age-related decline in survival rate and reproduction—nearly ubiquitous in the natural world? Or as George Williams put it, ‘it is remarkable that after a seemingly miraculous feat of morphogenesis, a complex metazoan should be unable to perform the much simpler task of merely maintaining what is already formed’ [1]. Why doesn’t evolution, in other words, mold the biology of organisms such that aging never occurs?

One possible solution to this conundrum is that evolution *does* in fact mold the biology of organisms such that they never age *in their natural environment*, that is, the environment in which they evolved. Aging might seldom occur in nature and only become evident when animals live much longer than they ever would in the wild, such as when we protect them from natural hazards by making them pets or livestock, keeping them in zoos or, as in the case of ourselves, organizing them into climate controlled, predator-free civilizations. Some biomedical gerontologists believe this hypothesis to be the case. But it is not and, in fact, dozens of field studies to date have identified that aging in wild animals is rampant if not close to ubiquitous [2].

Thus, there is a real puzzle to be solved as to how aging develops in natural populations. Fortunately, evolutionary biologists have cracked this mystery.

## EVOLUTIONARY THEORIES OF AGING

Sir Peter Medawar, one of the most influential fathers of evolutionary aging research, noted the similarities between the puzzle of aging and the puzzle of Huntington’s disease, an invariably fatal autosomal dominant neurological disease [3]. If the disease is inherited and always fatal why hasn’t natural selection expunged the responsible alleles from our gene pool? The answer, of course, has to do with the fact that Huntington’s disease typically strikes late—during, or even after, the child-bearing years. In evolutionary terms, a deleterious allele for health that does not affect the reproductive success of those who carry it is immune to the cleansing power of natural selection. In contrast, a mutant allele that compromised reproduction early in life would of course be strongly disfavored by natural selection. By this logic, the earlier in reproduction an allele’s effects were felt, the greater would be selection’s impact on its fate. The implication of this simple idea is that the power of natural selection to favor or disfavor new alleles wanes the later in life their effects are manifest.

Medawar’s insight was in realizing that the same logic could also be applied to aging. He posited that as new mutations are constantly occurring, and many more new mutations are deleterious rather than beneficial, any new deleterious mutation with effects on reproduction or survival that emerged only late in life would be insufficiently opposed by natural selection and such alleles could accumulate in the genome over generations. This concept/idea is referred to as Medawar’s mutation accumulation hypothesis.

George Williams accepted Medawar’s fundamental insight; however, he noticed something that was implicit in Medawar’s insight that Medawar himself mentioned only in passing. He pointed out that if there were alleles that had beneficial effects on survival or reproduction early in life when the intensity of natural selection was strong, but caused detrimental effects later on when its intensity had weakened, those alleles could be actively favored by natural selection despite their destructive late life effects [1]. The positive impact of an allele in early life versus its later negative effect has come to be called antagonistic pleiotropy—pleiotropy being the descriptive term for multiple effects of a single gene. A corollary of Williams’ hypothesis is that new mutations observed to lengthen life and slow aging are likely to have some detrimental impact on early life survival or reproduction. In addition to elaborating the general concept of antagonistic pleiotropy, Williams 1957 paper also made nine specific predictions about the existence of, and relative rates of, senescence under certain conditions. Sixty years later on, amazingly six of the nine predictions have at least limited empirical support [4].

But which theory of aging, mutation accumulation or antagonistic pleiotropy, is more pervasive across species? A little noticed implication of Medawar’s mutation accumulation hypothesis is that because mutations occur randomly, the ensemble of damaging mutations that accumulate in one genetic lineage will be idiosyncratic to that lineage and therefore will inevitably differ from those in another lineage. Consequently, the mechanisms underlying aging, assuming mutation accumulation is its chief mechanism, would be expected to differ even among lineages within a species much less across species. If on the other hand, we assume that a limited number of biological processes have the rather strange characteristic that they can be beneficial early in life yet detrimental later on, then Williams’ hypothesis of antagonistic pleiotropy suggests that many mechanisms of aging may be conserved across species. The modern use of model organisms to understand aging in general actually *presupposes* antagonistic pleiotropy, in other words. Is that presupposition warranted?

## TESTING EVOLUTIONARY THEORIES

Hypotheses about genetic mechanisms of aging are most often tested in the laboratory these days. One major advantage of laboratory tests is that the environment can be rigorously controlled. However, a major disadvantage of laboratory conditions for testing evolutionary hypotheses is that they generally do not even vaguely resemble the environment in which the species and traits in question actually evolved. The physical environment is constant and benign in the laboratory rather than unpredictably variable as in the real world. The biological environment, including the lack of predators, competitors and parasites, is also benign and invariant. Experimental results obtained in the laboratory need to be interpreted with caution when considering their evolutionary significance. To mention one example, certain hypomorphic mutations in the *daf-2* insulin-IGF receptor locus in the nematode *C. elegans* has been shown repeatedly to extend life and vigor [5–8], which raises the evolutionary question why has the wild-type allele not been replaced by one of these hypomorphic alleles in nature? The answer has been nicely answered by simply competing a long-lived mutant strain, initially reported to have normal growth and fertility, against a wild-type strain. The long-lived mutant disappears within a handful of generations due to a small, previously unnoticed, reduction in early life fertility [9]. Similarly, maintaining worms in soil, their natural habitat, rather than agar, their usual laboratory habitat, obliterates the survival advantage of a similar, long-lived, *daf-2* mutant [10]. These results demonstrate several reasons why evolutionary hypotheses are most rigorously assessed under natural, or at least semi-natural, conditions.

Evolutionary hypotheses *can* be informatively tested in the laboratory by imposing experimental evolution paradigms. These apply a specific selection regime, and evolutionary change over generations is monitored. For instance, Stearns and colleagues imposed high random mortality on one group of fruit flies and low random mortality on another for 60 generations and observed that the high mortality regime led to shorter life, reduced age of sexual maturity and an accelerated reproductive trajectory compared with the low mortality regime [11]. These results were strikingly similar to those found in a natural experiment in which opossums naturally evolving on a predator-free island were compared with opossums on a normal-predation mainland population [12].

Below we provide two examples of the antagonistic pleiotropy hypothesis in natural populations followed by more specific examples in each of the four commonly studied laboratory organisms: yeast, worms, flies and mice to show how laboratory model organisms allow us to discover individual antagonistically pleiotropic genes. Note that although antagonistic pleiotropic effects (i.e. trade-offs in reproduction and longevity) have been posited in humans, there are no compelling cases where the underlying gene or allele responsible for the trade-off has been identified. This is because studies of human genetics are observational rather than experimental and therefore rely on correlations which may or may not be causative. Therefore although we discuss below the implications for humans we do not enumerate what are necessarily speculative examples.

### Trade-offs in natural populations

For decades, ecologists and evolutionary biologists have noted dozens of trade-offs among life history traits in natural populations [13, 14], most revolving around ‘costs of reproduction’, in which individuals that mate (or mate more) have a corresponding decrease in some fitness trait whether it be immunity, energetic ability or longevity. Note that such trade-offs are *consistent* with the hypothesis of antagonistic pleiotropy, but because the genetic bases of trade-offs in natural populations are typically unknown, they cannot be cleanly attributed to that mechanism. We note in passing that these trade-offs are also consistent, indeed provide strong support, for Kirkwood’s disposable soma theory of aging [15] which is also compatible with antagonistic pleiotropy. The literature on trade-offs in natural populations is vast [13] and other reviews have integrated multiple facets of the costs of reproduction [16–18]. Here we highlight only a few examples that may have antagonistic pleiotropic bases: reproduction and longevity as well as reproduction and immunity.

#### Reproduction and longevity

The most commonly observed trade-off in natural populations involves describing negative associations between fecundity and longevity. Negative correlations between lifespan and reproduction have been described across numerous vertebrates including reptiles, birds and mammals [19–21]. In addition, trade-offs in invertebrates are widely reported, although usually in the laboratory context where wild caught animals can be individually studied (e.g. [22–24]). For example, within a large population of Western gulls, individuals that mate early in life have an increased risk of mortality compared with older first-time breeders [25]. Similarly, red squirrels that breed early have shorter lifespans than those that delay first breeding [26]. Interestingly, these trade-offs are often absent among captive mammals and birds, suggesting that the selective pressures that drive antagonistic pleiotropy are relaxed under the benign environmental conditions [27]. Thus, while trade-offs between lifespan and reproduction appear common in natural populations, because we most often do not know the individual genes involved, we cannot unequivocally attribute them to antagonistic pleiotropy as contrasted with, say, the disposal soma hypothesis.

#### Reproduction and immunity

While the trade-off between reproduction and longevity or other measures of aging are the most commonly studied ‘costs of reproduction’, immune function has also been shown to be negatively affected by early life reproduction. In a pathogen-rich environment, compromised immune function would be expected to shorten life expectancy. For instance, female common eider sea ducks that raise larger clutches have decreased immune function that *may* decrease future survival as well as reduce future reproduction of those that do survive [28, 29]. Along the same lines, mating in the striped ground cricket suppresses aspects of immunity driving increased mortality in mated individuals [30]. In addition, *Drosophila* males mated to higher numbers of females have decreased ability to clear bacterial infections [31]. Finally, trade-offs have also been described between reproduction and the ability to resist parasitic infections in collared flycatchers, thus indirectly affecting longevity [32]. All of these trade-offs between reproduction and immunity are potentially driving other trade-offs in reproduction and longevity, and as such are potentially mediated by antagonistic pleiotropy genes.

### Antagonistic pleiotropy in the laboratory

While trade-offs between reproduction and other fitness components appear to be common, possibly even ubiquitous throughout natural populations, individual genes that are causing these trade-offs are rarely investigated for multiple reasons including lack of genomic resources for many species and not being of general interest to those ecological researchers interrogating natural populations. The actual genes that contribute to antagonistic pleiotropy have most commonly been determined in model organisms in the laboratory. To this end, the focus of this brief review will turn to antagonistic pleiotropic effects of specific genes that have been discovered in the four commonly studied laboratory models: yeast, worms, flies and mice. The discovery of antagonistic pleiotropy in the laboratory has occurred by either (i) direct selection experiments for long life or (ii) partial or full inactivation of a gene leads to a significant increase in lifespan; however, when investigated further, this alteration has had a deleterious effect on some component of early life fitness (Table 1). Table 1 summarizes several well validated genes that have been discovered by the latter in common model organisms.

**Table 1.** Selected examples of genes when mutated in the laboratory lead to lifespan extension with antagonistic pleiotropic effects

Species	Gene	Longevity increase	Pleiotropic effect
*C. elegans*			
	*daf-2*	100%	Reduced early life reproduction
	*age-1*	65%	Reduced starvation resistance
*D. melanogaster*			
	*chico*	50%	Sterility
	*Inr*	85%	Sterility
*Mus musculus*			
	*prop-1*	50%	Sterility
	*p66^shc^*	35%	Reduced fecundity and maternal behavior

#### Yeast

Unicellular budding (or Brewer’s) yeast (*Saccharomyces cerevisiae*) is one of the most studied aging models, and as such, many genes that have antagonistic effects have been discovered in yeast. For instance, 65% of all yeast strains with extended replicative lifespan due to the ablation of single genes have lower fitness than wild-type yeast in direct competition experiments [33]. The majority of the decreased fitness is due to reduced growth rate relative to wild-type. In addition, previous systems biology analyses suggest that antagonistic pleiotropy may be a significant contributor to protein–protein interaction networks and network connectivity [34]. In another yeast species, the fission yeast, *Schizosaccharomyces pombe*, chromosomal rearrangements can lead to decreased reproduction (meiosis) but increased growth (mitosis) in antagonistic pleiotropy fashion [35].

#### Worms

The roundworm, *Caenorhabditis elegans*, is the most studied invertebrate model in aging research. As such, work in the worm has discovered hundreds of genes that extend life when suppressed [36]. However, studies of these long-lived mutants often fail to indicate whether there are any reproductive effects either in fecundity or the timing of egg laying. Yet when they have been seriously looked for, antagonistic pleiotropic effects have usually been discovered.

As mentioned in the introduction, the most robust gene with the greatest positive impact on lifespan in *C. elegans* is *daf-2*, the worm insulin/IGF receptor. Hypomorphic mutations in this gene show a significant decrease of 18–23% in reproduction compared with wild-type worms while doubling lifespan. The reproductive impact of the longevous *daf-2* mutation may seem fairly mild for a gene that doubles lifespan, however the reproductive effects occur early in life when selection is strong [7]. When directly competed against one another, *daf-2* mutants were quickly outcompeted by wild-type worms. Under constant feeding conditions *daf-2* mutants were extinct within four generations and with pulsatile food availability they became extinct even quicker—within three generations [8]. Hypomorphic mutations in the *age-1* gene, also in the insulin/IGF signaling pathway, result in 65% longer life compared with wild-type worms [37]. Interestingly, when directly competed against wild-type worms in the laboratory with abundant food, neither appeared to have an advantage. However when food is only episodically available, likely the pattern in nature, *age-1* quickly goes extinct [38], suggesting why the wild-type allele persists in nature.

One other example is the *C. elegans clk-1* gene, which produces an enzyme required for ubiquinone biosynthesis. When *clk-1* is genetically disrupted, longevity is extended from 20% to 40%, depending on temperature. There are also multiple early life antagonistic pleiotropic effects, including reduced metabolic activity, prolonged development and reduced reproductive rate [8]. Along similar lines, mutations in 24 developmental genes that extend life also decrease fecundity. Many of these genes, like *clk-1,* are working outside of the well-described insulin/IGF-signaling pathway [39].

In combination, these results suggest that there can be very different pleiotropic effects of lifespan-extending genetic interventions, and while not all affect reproduction directly, some component of early life fitness is inevitably reduced, which would contribute to a lack of selection for the longevous allele under normal conditions.

In addition to individual genes that have been knocked out, a selection experiment in worms has provided insights into the ubiquitous role of antagonistic pleiotropy in life history trade-offs. When early reproduction was selected for more than 40 generations, a trade-off in late reproduction was observed, but shortening of lifespan was not. This experiment was done under laboratory conditions, so it should be interpreted with caution. However, it does indicate that although antagonistic pleiotropy constraints appear ubiquitous, they may not always involve longevity. They could involve reproductive longevity as well [40].

**Figure 1.**
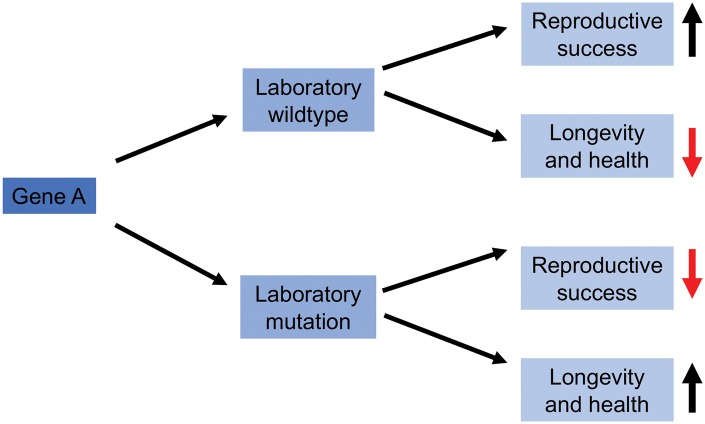
Basic model to describe how laboratory mutations lead to lifespan extension with reductions in reproductive success

#### Fruit flies

Species of the fruit fly genus *Drosophila* are probably the most studied species group for investigating *evolutionary* questions about aging. Experiments in the fruit fly have ranged from empirical studies on the longevity consequences of selection for delayed reproduction in the laboratory [41, 42] or more commonly these days discovery of genes that increase lifespan [43–45]. Dozens of genes have now been discovered that when inactivated or overexpressed increase longevity in the most commonly used fruit fly species, *D. melanogaster* [36]. However, as is seen with worms, many reports on the longevity effects of these mutations neither report reproductive or other components of early life fitness effects nor have they been replicated in multiple laboratories. For this reason, we will focus on some of the most reproducible individual gene mutations as well as a few of the many selection experiments carried out in *D*. *melanogaster.* We note that a summary of correlated responses to selection in *Drosophila* laboratory selection may be found in [46].

Experimental evolution of selected lines and hybrids suggest that antagonistic pleiotropy rather than Medawar’s mutation accumulation is the major genetic mechanism driving late life mortality in flies [47]. *D. melanogaster* lines selected for later life reproduction live longer with lower early life reproduction than controls [48]. In addition, a negative correlation is seen between early life reproduction and starvation resistance—a commonly measured trait that decreases with aging in *Drosophila* [49]. Similarly, *D. simulans* lines selected for delayed growth have increased longevity, again suggesting antagonistic pleiotropy, but delayed maturity did not cause an overall decline in fecundity as might have been expected [50]. However, delayed growth in the wild would potentially lead to negative fitness consequences and is thus selectively disadvantageous. A second set of *D. melanogaster* lines selected for early or late reproduction found longer life in the late reproducing lines but no decrease in early reproduction [51]. However, larvae from the shorter-lived, early reproducing lines were competitively superior to larvae from the old reproducing lines. This suggests antagonistic pleiotropy trade-offs between larval growth and longevity, not reproduction and longevity, emphasizing the fact that there are multiple important components of early life fitness. Interestingly, experiments of multiple, independent artificially selected lines show that the laboratory conditions can have large effects on life history traits, especially fecundity. This would suggest that the effects of antagonistic pleiotropy genes are strongly affected by the environment such that one laboratory may show a longevity-reproduction trade-off while others do not, even in the same lines [52].

Some studies in *Drosophila* find co-occurrence of evidence for antagonistic pleiotropy and mutation accumulation theories [53, 54], suggesting—as should be apparent—that these two potential evolutionary mechanism of aging are not mutually exclusive. Some interesting recent work across a range of inbred *Drosophila* lines, suggests that many single nucleotide polymorphisms are associated with both antagonistic pleiotropy and mutation accumulation in response to several stressors [55]. While both antagonistic pleiotropy and mutation accumulation may contribute to life history traits in *Drosophila*, it should be noted that all studies of evolution in the species group appear to find at least one antagonistic pleiotropy trade-off.

As has been described in yeast and worms, individual genes have also been shown to extend longevity with negative effects on reproduction in *Drosophila melanogaster*. As in worms, numerous genes having such antagonistically pleiotropic effects in *Drosophila* are involved in the insulin/IGF signaling pathway. For instance, disabling the *Drosophila chico* gene, the fly insulin receptor (InR) substrate gene, increases longevity with a corresponding decrease in fertility in heterozygotes. Homozygote mutant females are long-lived and sterile [45]. Knockdown of the *Drosophila* InR itself increases longevity, but females do not produce viable eggs, potentially through its impact on juvenile hormone expression [44]. And direct knockdown of juvenile hormone itself also increases lifespan with a significant reduction in female egg production [56]. However, reduced activity of all insulin-signaling genes does not have direct effects on fertility. For instance, overexpression of *Drosophila* forkhead transcription factor (dFOXO) extends life with no obvious effect on fecundity [57]. Similarly, long-lived *indy* [58] and *jnk* [59] mutant flies do not show any costs of reduced fertility associated with increased lifespan. However, as noted previously, there are a number of early life fitness components besides overall fertility. Thus, trade-offs cannot be ruled out. For instance, very few researchers interested in aging examine larval competitive ability, even though it has been shown in several studies to involve a trade-off with adult longevity [51]. In fact, in several independent lines of long-lived flies produced by artificial selection, the most consistent trait associated with long life was reduced larval viability [60]. Surprisingly then, a number of genes that have been discovered to increase lifespan in *Drosophila* without obvious fertility effects, but then again fertility may not be the chief antagonistic trade-off with long life in flies. To this end, we would not be surprised to learn that most lifespan extending genes confer a negative effect on other aspects of overall fitness.

#### Mice

In house mice (*Mus musculus*), neither field studies nor focused long-term laboratory selection studies—the preferred ways to evaluate evolutionary hypotheses—have been purposefully done. However, because laboratory mice have been selected over many generations for high reproductive rate and other traits associated with their commercial use in laboratory research, something of a ‘natural experiment’ has been performed. Under laboratory conditions, domesticated mice have accelerated reproductive maturation and larger litters compared with wild-derived mice (recent descendants of wild mice living in the laboratory) [61, 62]. Given their selection for accelerated reproduction, according to antagonistic pleiotropy, one might expect laboratory mice to age more quickly than wild mice as a consequence. Indeed, at least under laboratory conditions this turns out to be the case [61].

One other mouse study under quasi-field conditions deserves mention. Ablation of the stress- and growth-factor responsive, cytoplasmic signal transduction protein *p66^shc^*, one of three splice variants coded by the *shc* locus, was originally reported to extend life and to exhibit increased cellular resistance to oxidative stress in mice [63]. Although a follow-up study could not replicate the life extension effect, other health-enhancing features discovered in the *p66^shc^* knockout mouse include enhanced insulin sensitivity as well as resistance to obesity, atherosclerosis and ischemic injury [64]. Again, under the antagonistic pleiotropy hypothesis, these health-promoting features associated with deficiency in normal gene products would be predicted to have negative fitness consequences. Indeed, when wild-type laboratory mice and mice either fully knocked out or heterozygous for *p66^shc^* were released into a large outdoor enclosure in western Russia for 13 months, the wild-type genotype increased nearly 3-fold in numbers, the knockout mice decreased in numbers by 4-fold and the number of heterozygous mice remained relatively constant. Further laboratory investigation suggested possible reasons for the poor performance of the mice with one or two *p66^shc^* alleles knocked out included worse cold- and starvation-tolerance as well as reduced fecundity and maternal behavior [65]. So, both the full knockout and the haplo-insufficient genotypes had reduced Darwinian fitness—at least in this environment—compared with wild-type. This, again, is consistent with antagonistic pleiotropy associated with the *p66^shc^* gene.

There are now more than two dozen engineered mouse genotypes that increase longevity under laboratory conditions. However, developmental or reproductive parameters are seldom noted for mice engineered for retarded aging or increased longevity. Perhaps best characterized in reproductive terms are the Ames and Snell dwarf mice because some early studies investigated their developmental and reproductive features prior to it being discovered that they were exceptionally long-lived. Ames dwarf mice display the largest longevity increase of any known mouse mutation and have a single disrupted transcription factor (*prop-1*) that is critical for the development of the pituitary. Consequently, they are deficient in several pituitary hormones (growth hormone, prolactin and thyroid-stimulating hormone), which makes them small, cold-sensitive and sterile. Snell dwarf mice lack a transcription factor (*pit-1*), which is activated by *prop-1*. It has nearly an identical phenotype to the Ames dwarf and both have multiple features resembling slowed aging. In addition, both genotypes are long-lived, with Ames dwarfs living 48–67% longer than controls [66] and Snell dwarfs living 42% longer than controls [67]. Both dwarf genotypes also have reduced fertility or sterility associated with their longevity [68]. Clearly, this could be interpreted as antagonistic pleiotropy under laboratory conditions, and this mutation would never be selected for in wild populations.

## CONCLUSION

When George Williams first proposed his theory of antagonistic pleiotropy in 1957, he concocted a hypothetical example of a gene that hastened the calcification of arteries during development but led to the calcification of arterial walls in later life. He did this because there were no genes known to have the rather odd characteristic of being beneficial in early life but detrimental later on. In the past 20 or so years, molecular biology has presented us with a cornucopia of such genes, and of nine predictions Williams made about senescence and aging, six have proven correct over the last six decades [4]. Ironically, it is the other sort of antagonistic pleiotropy, late life benefits at the expense of early life decrements that are now in the forefront of aging research.

We have barely touched the surface of a very large topic here. But it is a topic that should have increasing interest moving forward because aging biology is on the verge of some major advances. From current evidence, antagonistic pleiotropy is somewhere between very common or ubiquitous throughout the animal world (and though not discussed here, except briefly in yeast, potentially all living domains). Whenever an allele or new mutation is discovered to extend life, some detrimental effect on early life fitness is almost always observed. This explains why longevity alleles are not favored in wild populations. However, while antagonistic pleiotropy appears to be nearly ubiquitous, a majority of actual antagonistically pleiotropic alleles remain undiscovered in natural populations, and laboratory studies that describe alleles that extend lifespan often do not report early fitness effects. These gaps in our knowledge still need to be addressed, and we predict that results from both will provide even more compelling evidence for the antagonistic pleiotropy theory of aging. One finding that has emerged in recent years that is particularly reassuring for those of us seeking medical interventions to prolong human health is that such interventions often do not have to be deployed until relatively late in life—after reproduction is finished [69]. In these cases, whether there would have been antagonistically pleiotropic side effects in early life is moot. Thus, evolutionary biology does not preclude the possibility of medical interventions in the aging process.

## FUNDING

We are grateful for funding support from a Glenn/American Federation for Aging Research postdoctoral fellowship (J.M.H.) and the U.S. National Institutes of Health grants P30 AG050886 and R01 AG057434 (S.N.A.).

**Conflict of interest**: None declared.
